# A Video Mosaicing-Based Sensing Method for Chicken Behavior Recognition on Edge Computing Devices

**DOI:** 10.3390/s24113409

**Published:** 2024-05-25

**Authors:** Dmitrij Teterja, Jose Garcia-Rodriguez, Jorge Azorin-Lopez, Esther Sebastian-Gonzalez, Daliborka Nedić, Dalibor Leković, Petar Knežević, Dejan Drajić, Dejan Vukobratović

**Affiliations:** 1 Department of Computer Science and Technology, University of Alicante, 03690 San Vicente del Raspeig, Alicante, Spain; jazorin@ua.es; 2 Department of Ecology, University of Alicante, 03690 San Vicente del Raspeig, Alicante, Spain; esther.sebastian@ua.es; 3 DunavNet DOO, Bulevar Oslobođenja 133/2, 21000 Novi Sad, Serbia; dalivorka.nedic@dunavnet.eu (D.N.); dalivor.lekovic@dunavnet.eu (D.L.); petar.knezevic@dunavnet.eu (P.K.); ddrajic@etf.bg.ac.rs (D.D.); 4 Paviljon Računskog Centra, The Department of Telecommunications, School of Electrical Engineering, University of Belgrade, Bulevar kralja Aleksandra 73, 11120 Belgrade, Serbia; 5 Faculty of Technical Sciences, University of Novi Sad, Trg Dositeja Obradovića 6, 21000 Novi Sad, Serbia; dejanv@uns.ac.rs

**Keywords:** chicken behavior recognition, convolution neural networks, mosaic images, mosaic videos, edge computing

## Abstract

Chicken behavior recognition is crucial for a number of reasons, including promoting animal welfare, ensuring the early detection of health issues, optimizing farm management practices, and contributing to more sustainable and ethical poultry farming. In this paper, we introduce a technique for recognizing chicken behavior on edge computing devices based on video sensing mosaicing. Our method combines video sensing mosaicing with deep learning to accurately identify specific chicken behaviors from videos. It attains remarkable accuracy, achieving 79.61% with MobileNetV2 for chickens demonstrating three types of behavior. These findings underscore the efficacy and promise of our approach in chicken behavior recognition on edge computing devices, making it adaptable for diverse applications. The ongoing exploration and identification of various behavioral patterns will contribute to a more comprehensive understanding of chicken behavior, enhancing the scope and accuracy of behavior analysis within diverse contexts.

## 1. Introduction

Understanding chicken behavior in the context of animal welfare monitoring provides crucial information for evaluating the flock’s health. This involves observing activities such as eating [1], drinking [2], resting [3], stretching [4], walking [5], running [6], preening [7] and others, like chicken being unhealthy [8]. The importance of deviations from the standard behavior of chickens cannot be overstated, as they serve as crucial indicators of possible problems or illnesses within the flock. These variations are effectively utilized to detect health problems and infections. Early identification enables immediate action to be taken, effectively halting the spread of illness and enhancing the overall health of the flock [9].

Farmers can enhance farm management [10] by gaining a comprehensive understanding of chicken behavior. This knowledge enables them to improve various aspects such as living conditions, feeding schedules, and stress management techniques. By optimizing these factors, farmers can achieve increased productivity and make effective use of resources [11]. Additionally, certain behaviors exhibited by chickens can serve as indicators of inefficient resource utilization [12]. This insight encourages the implementation of methods like optimal feeding and waste reduction, further promoting efficient resource management on farms [13].

In the field of precision livestock farming [14], the recognition of chicken behavior holds immense importance [6]. This technology-driven approach focuses on precise monitoring and management of individual chickens or groups, leading to enhanced efficiency and sustainability in poultry farming. Furthermore, monitoring and comprehending chicken behavior contribute to the implementation of ethical farming practices. By aligning the farming environment with the normal behaviors of chickens, farmers can create conditions that promote improved welfare and better living standards [9,15,16].

Assessing the well-being of chickens heavily relies on monitoring their behavior, which proves beneficial for farmers and researchers alike. By closely observing bird species and their activities using automatic recognition technologies [17], valuable insights can be gained regarding the overall health and happiness of the flock, contributing to the understanding of diverse emergent collective behaviors [18]. This knowledge enables early detection of health issues and diseases [6,19], leading to timely intervention and improved flock health [3].

Detecting chicken behavior is crucial for animal welfare [9], health monitoring, farm management, and sustainable farming, but current methods struggle with scalability, real-time processing, and edge device performance [20].

Traditional methods of animal behavior recognition frequently rely on manual observation [21] or centralized data processing systems, which may be time-consuming, labor-intensive, and expensive. Furthermore, these strategies may fail to deliver the real-time insights required for fast intervention and decision-making. Recent advances in machine learning and video analysis provide potential alternatives, but they frequently need significant processing resources, rendering them unsuitable for implementation in resource-constrained contexts common to many farms.

In view of these obstacles, our research presents an innovative approach to identifying chicken behavior utilizing edge computing devices. This solution uses visual sensing mosaicing and deep learning to perform accurate behavior detection at the edge, decreasing latency and dependency on cloud-based processing. Edge computing devices, with their close proximity to data sources and minimal power consumption, are a perfect alternative for real-time monitoring and analysis in agricultural settings.

Within this study, we present a method for identifying chicken behavior on edge computing devices using video mosaicing, which distinguishes chicken behavior by fusing deep learning techniques with the process of creating pictures from several sequential video frames. Our solution combines video sensing mosaicing—a technique for stitching together video frames to provide a comprehensive view of the environment—with the powerful capabilities of deep learning models. Using MobileNetV2 as the backbone, a lightweight convolutional neural network, we achieved a 79.61% accuracy rate for three different types of behavior in our testing. This method is advantageous due to its simple architecture, making it easier to implement and understand, and its suitability for complex tasks with long dependencies, as well as tasks requiring modeling of both long-term and short-term dependencies.

Our work is significant not just for technological innovation, but also for its practical ramifications. Our technique promotes proactive and informed farm management [22] by allowing efficient behavior recognition on edge devices, resulting in enhanced animal care and operational efficiency. Furthermore, this study sets the path for further exploration and development of behavioral analytic methodologies, enabling a better knowledge of chicken behavior in a variety of circumstances.

Furthermore, our research fills a major need in the field of poultry farming [23] by developing a scalable, real-time, and accurate method for recognizing chicken behavior on edge computing devices. This development has the potential to change present practices and accelerate progress toward more sustainable and compassionate agricultural methods.

The paper begins with an introduction (see Section 1) to the problem of recognizing chicken behavior on edge computing devices, presenting a technique involving video mosaicing and deep learning. It highlights the achieved improvements in accuracy. The materials and methods section (see Section 2) describes the materials used, including edge computing devices and datasets, and explains the video mosaicing and deep learning methods employed, along with details of the experimental setup. In the experiments and results section (see Section 3), the experimental findings, including accuracy metrics, are presented. This section further analyzes the results, discusses encountered challenges, and suggests future research directions. Finally, the conclusions section (see Section 4) summarizes the study’s objectives and accomplishments, emphasizing the significance of the proposed technique and providing closing remarks on the implications and potential impact of the research.

## 2. Video Mosaicing Method to Classify Chicken Behavior

This section provides an overview of the structure of our method, which involves the utilization of videos obtained from Closed Circuit Television (CCTV) cameras deployed in a poultry farm environment. A camera was installed above the chickens in the cage capturing 360-degree videos. To ensure comprehensive coverage, wide-angle lenses were utilized to encompass the entire area, including the cage, within the Field-of-View (FOV).

The first variable that is very important to mention is the mosaic image division pattern. Let us explain how mosaic images are constructed and how they are created from the videos. Mosaic images are matrix-like structures made of sequential images cropped from video frames. Each of these cropped images contain one specific chicken performing one activity at a time. Coordinates and dimensions of each of the cropped images of chickens in the frame is called a ’bounding box’ and has only one chicken pictured in it. Mosaic images, when created, already have annotations attached to them, indicating what type of activity a specific chicken is performing and what the are parameters of each bounding box. Ornithologists or data scientists use annotation tools to label chicken activities. When labeled, the information about which frames’ bounding boxes was saved into an annotation file.

Another important variable is video data sampling, which is measured in frames per second, or FPS. This is an indicator for video sequences, as well as for mosaic images. In case of videos, this number means how many frames are shown to a viewer of a video per second. For example, if a video is recorded with a 20 FPS frame rate, in one second, a viewer will see exactly 20 frames of this video. In case of mosaic images, the FPS number means how many frames of the original video it needs to skip in order to form a mosaic image using a division pattern made from consecutive bounding boxes.

In our case, it did not work well, and we were missing a lot of data in the final dataset, so we decided on a different approach. We used a variable computed from the video FPS divided by the mosaic FPS, indicating how many frames we need to shift our focus relative to the position of the first frame of the video so that another sub-image could be included in the mosaic image. For example, the following frames could be selected: 21, 41, 61, 81, *…*, 5981. In another cycle, the frames could be 22, 42, 62, 82, *…*, 5982, which will form mosaic images, and so on.

If the FPS of a video needs to be lowered to match the required FPS of mosaic images, a conversion needs to take place based on the following formula:

(1) $$FR_{i}=\left\lfloor \frac{FPS_{o}}{FPS_{t}}\right\rfloor$$

where $FR_{i}$ is a frame interval, with *i* being a number of resulting frames-per second “shifts” relatively to the first frame of a video sequence; $FPS_{o}$—a number of frames-per second of the original video sequence; and $FPS_{t}$—a number of frames-per second of the target mosaic image.

Being of the temporal nature, the video mosaicing method used for bird behavior recognition relies on the frequency of video frames data sampling. For example, for slower moving objects, or objects not moving at all, like sleeping chickens, a high sampling rate is not necessary.

**Figure 1:**
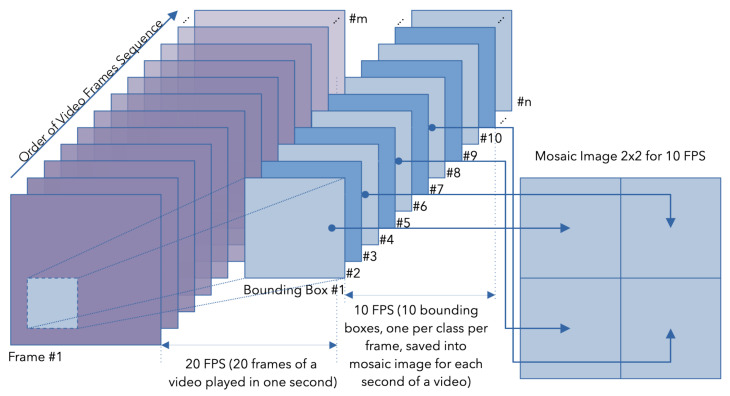
Image mosaicing process: converting cropped chicken video frames (count #m) into bounding boxes (count #n) then into mosaic images (one mosaic image 2 × 2 matrix where chickens are captured at 10 FPS); here, the class is to be understood as a Chicken ID.

In the field of bird behavior recognition, video data sampling is an important factor, specifically when using the video mosaicing technique. Because this is a temporally intensive task, the identification accuracy is highly dependent on how frequently video frame data are sampled. When dealing with slower-moving objects or situations where things stay still, as when you are observing sleeping chickens, you need to modify the sampling rate, which is commonly expressed in FPS. In these situations, we can ensure that we make better use of computing resources while still successfully capturing essential behavioral details by lowering the FPS. The process of retrieving video frames and converting them into mosaic images is described in Figure 1.

In this picture, the bounding boxes 1, 3, 5, and 7 are taken from the Frame 1, which originates from the video sequence, and in this order construct, the mosaic image is shown on the right side. The process continues with bounding boxes 9, 11, 13, and 15, etc., but this time they are used to create another mosaic image, which is second in order. Other frames or bounding boxes are not considered until the process is repeated for another shift from the beginning of the bounding boxes in the video sequence while simultaneously preserving the frame rate in the mosaic images.

**Figure 2:**
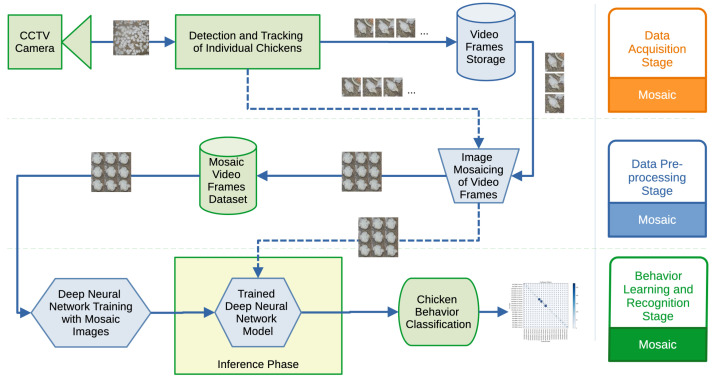
System Architecture: The diagram illustrates visual data acquisition from a CCTV camera, further data pre-processing and creation of mosaic images from the videos, and finally training of the Deep Neural Network in the behavior learning and recognition stage with the inference phase.

The architecture designed for behavior learning and recognition is illustrated in Figure 2. In the following subsections, we will explain each of the architectural components.

### 2.1. CCTV Camera Input

In the data acquisition stage, a CCTV camera captures video footage, which is initially stored on-site at the chicken farm and then transferred to a cloud environment. This environment utilizes cloud computing technologies and services for data storage and processing.

### 2.2. Detection and Tracking of Individual Chickens

At this stage, individual chickens are detected and tracked in the captured video file. The detection was tested using the YOLOv8 algorithm and multi-object tracking using the SMILEtrack model. Additionally, other options such as UCMCSTrack, PPTracking, MOTRv2, Deep-EIoU, GLEE-Pro, MVFlow, ReMOTS, GSDT, GSTrack, and various other models are available. Another approach involves real-time data processing, indicated by a dashed line from the “Detection and Tracking of Individual Chickens” stage through the “Image Mosaicing of Video Frames” functionality to the “Inference Phase”, where a Deep Neural Network Model is trained. In this study, only the offline option for chicken behavior recognition was utilized.

### 2.3. Video Frames Storage

After receiving the video file for data processing, we detect individual chickens in each frame depicting the flock, track them, extract bounding boxes related to these chickens in the video, and subsequently use images of the chickens from these bounding boxes to train a deep neural network for behavior analysis. Later, this behavior is interpreted during the inference phase.

### 2.4. Image Mosaicing of Video Frames

The regular chicken images obtained from video frames undergo a video mosaicing algorithm, resulting in the generation of mosaic images. The distinction between image mosaicing and video mosaicing lies in their methodology; while image mosaicing arranges all regular bird images randomly into a structured mosaic image, video mosaicing utilizes sequential frames. This process may introduce delays between captured frames or duplicate frames, starting from a frame shifted relatively to the first frame of a video. These adjustments allow for the capture of a longer span of chicken behavior.

This method distinguishes itself from the mosaic data augmentation approach used in YOLOv4 [24], which generates composite images by arranging individual, cropped, and non-cropped images into rectangular grid sections. This approach may sometimes include their ground truths, using Mixout, Cutout, and CutMix algorithms.

In contrast, for our behavior dataset, we employed a mosaic method aligning frames of a chicken video vertically and horizontally into a matrix structure. This approach, referred to as the mosaic method, offers more comprehensive information about chicken behavior within a single mosaic image. These mosaic images are then stored in a dataset and utilized for training deep convolutional neural network models until satisfactory results are achieved.

The video mosaicing algorithm localizes multiple instances of the same chicken species in a given video. To extract images of chickens for training deep neural networks, we utilized the Computer Vision Annotation Tool (CVAT) [25]. Initially, we obtained CCTV video data from the chicken farm. Subsequently, we imported this data into the CVAT tool and configured it to recognize only one bird species—a chicken—as the annotation object, with multiple behavior categories (refer to Table 1). In OpenCV semi-automatic mode, we annotated each chicken’s behavior by enclosing each chicken instance in a bounding box and selecting the appropriate behavior class based on the chicken’s actions in the video. Once the video was fully annotated, we exported the annotations in COCO [26] 1.0 format as a JSON file. This file will later be read by our application, and all relevant information for each chicken instance will be converted into mosaic image structures and stored in a Mosaic Images Dataset. This dataset will be used to train our deep neural network methods for behavior classification.

In case of real-time video processing mosaic images are used in the inference phase for training of deep neural network model.

### 2.5. Mosaic Video Frames Dataset

Once mosaic images are created from video frames, they are stored in a dataset of mosaic video frames. These frames are then utilized for training, validation, and testing of chicken behavior. In our case, the dataset comprises videos constructed from mosaic images.

### 2.6. Inference Phase

In the next step, the trained Deep Neural Network model is employed for inference, providing classification results for chicken behavior as output. Subsequently, a confusion matrix depicting probabilities of behaviors is generated.

### 2.7. Final Thoughts on the Architecture

Our strategy leverages the power of convolutions, which are fundamental to CNNs. By applying a uniform filter across a mosaic image, we can identify specific features throughout the entire input image at various levels of detail. This remarkable capability, known as translational invariance, focuses on detecting the presence of features associated with particular behavioral patterns.

## 3. Experiments and Results

### 3.1. Mosaic Image Division Pattern

The matrix-like structure of mosaic images could have various formats. For example, we start with the division 2 × 2. In this format, there are four chicken images in the mosaic image—two at the top and two at the bottom, sequentially copied from bounding boxes and placed into the mosaic image from left to right and from top to bottom. As we continue with division patterns, such as 3 × 3, 4 × 4, 5 × 5, and 6 × 6, we increase the number of sequential chicken images in one mosaic image, aiming to increase the amount of behavioral information captured in each mosaic image, but at the same time, we reduce the amount of detail captured in each of the mosaic sub-images.

### 3.2. Video Sampling for Mosaic Images

The dimensions of mosaic images are standardized at 224 × 224 × 3, encompassing the RGB color space. Notably, each mosaic image possesses a relatively compact size, approximately 100 kilobytes, ensuring efficient data storage and processing. This approach leverages the richness of information embedded in mosaic images to enhance the precision and effectiveness of behavior recognition across different classes.

### 3.3. Describing the Dataset

Our newly devised dataset, which adopts an image mosaic format [20], is employed in conjunction with CNN models. By arranging frames into a matrix structure, we have created mosaic images that provide a more comprehensive understanding of the chickens behavior featured within the same video input. Our approach involves the utilization of convolutions, a fundamental attribute of CNN architectures, and includes the amalgamation of multiple images depicting a single chicken taken from video frames. Through training and testing various models on the mosaic dataset, our aim is to identify an efficient yet effective approach for chicken behavior classification.

**Figure 3:**
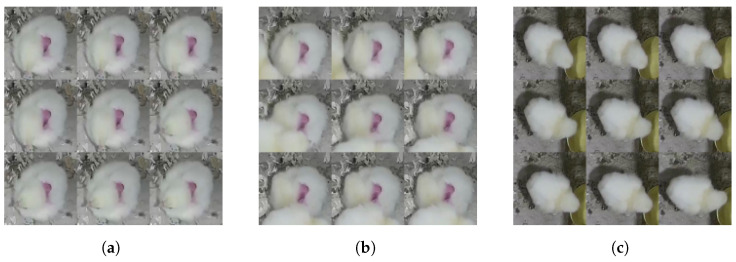
Mosaic images of chicken showing different behaviors, such as “Sleepy” and “Drinking”. (**a**) The Mosaic image of chicken No.1 showing “Sleepy” behavior, where the chicken is not changing its position, but sometimes moves with its head left and right observing its surrounding. (**b**) The mosaic image of chicken No. 1 showing “Sleepy” behavior while another chicken passes by and disturbs it, causing a change in body position. (**c**) The mosaic image of chicken No. 2 showing “Drinking” behavior, where the chicken approached the drinking pot.

In Figure 3, three pictures display two behaviors observed for chicken ID numbers 1 and 2. The “Sleepy” behavior does not imply that the chicken is completely still; rather, it indicates that the movement is not initiated by this chicken but by other factors. In the second image, it is evident that another chicken caused the disturbance, resulting in slight movement from the “Sleepy” chicken without altering its behavior.

**Figure 4:**
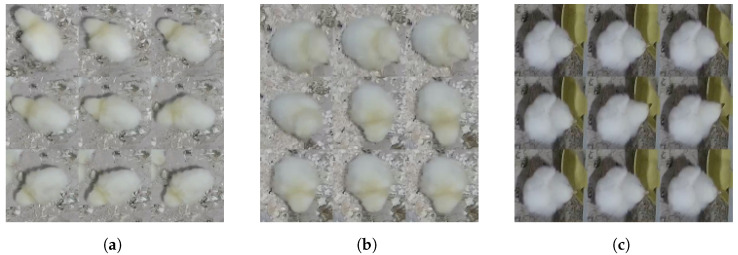
Mosaic images of chicken showing different behaviors, such as “Eating” and “Drinking”. (**a**) The mosaic image of chicken No. 2 showing the “Eating” behavior. (**b**) The mosaic image of chicken No. 2 showing the “Eating” behavior as in the (**a**), but in a different place in the cage. (**c**) The mosaic image of chicken No. 5 showing the “Drinking” behavior at the same place as chicken No. 2 from Figure 3c.

In Figure 4, three mosaic pictures depict two behaviors, “Eating” and “Drinking”, across three situations. In the first case (Figure 4a), chicken No. 2 is showing the “Eating” behavior, while consuming food found on the ground. In the second image (Figure 4b), the same chicken is seen consuming food in a different spot within the cage. Both mosaic images exhibit slight variations in the chicken’s movements and body part positions. In the third image (Figure 4c), chicken No. 5 is shown drinking from a water pipe positioned above a bowl.

**Figure 5:**
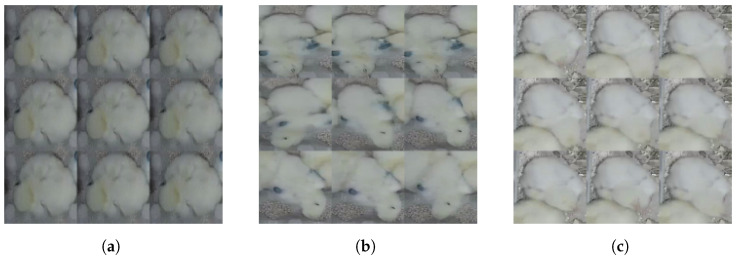
Mosaic images of chickens showing different behaviors such as “Sleepy” and “Eating”. (**a**) The mosaic image of chicken No. 7 showing the “Sleepy” behavior. (**b**) The mosaic image of chicken No. 11 showing the “Sleepy” behavior. (**c**) The mosaic image of chicken No. 12 showing the “Eating” behavior.

Another behavior we were able to observe is “Sleepy”, where chickens (ID No. 7 and No. 11) show a lack of motion in comparison to the other chicken (ID No. 12—see Figure 5). Generally speaking, each of the nine images in the case of the “Sleepy” behavior show less movement than in the case of the “Eating” behavior. Detecting disparities may prove to be a complex endeavor for a human specialist, yet the utilization of automation methods undeniably enhances the accuracy of this process.

### 3.4. Overview

We summarize our experimental findings and offer justifications for each method’s results in this section.

This section provides an overview of the structure of our evaluation model, which involves the utilization of videos obtained from CCTV cameras deployed in the poultry farm environment. In the case of a Serbian farm, a camera was installed above the chickens capturing 360-degree videos. Similarly, in the case of a farm in The Netherlands, a camera was positioned above the chickens in the cage. To ensure comprehensive coverage, wide-angle lenses were utilized to encompass the entire area, including the cage, within the Field-of-View (FOV).

**Figure 6:**
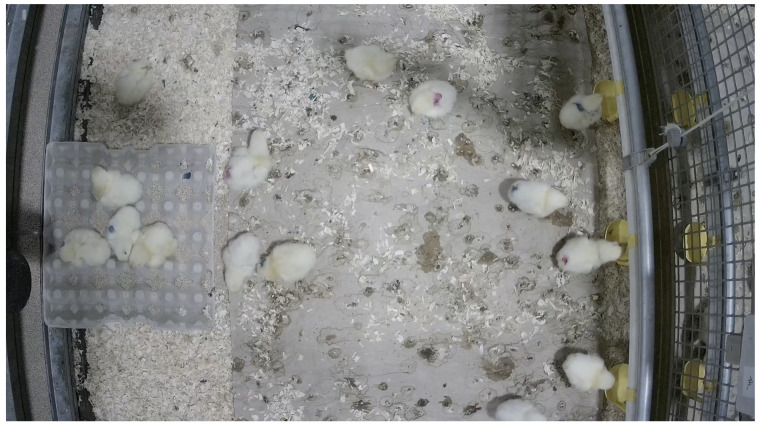
The picture displays a sample view captured by a fish-eye CCTV camera on a farm in The Netherlands.

In the course of this paper, we had the unique opportunity to annotate videos featuring chickens sourced from a farm located in The Netherlands (Figure 6).

In our research, we reviewed and converted 5 min video from a farm in The Netherlands, into a collection of mosaic images illustrating chicken behavior. This collection was then divided into three sets: training, validation, and testing, with a distribution ratio of 60/20/20. Afterwards, we utilized the mini-batch gradient descent algorithm, employing a batch size of 32 and a learning rate of 0.0001, to train the models. In order to address overfitting, we implemented a five-fold cross-validation approach for effective hyperparameter optimization. Our evaluations were conducted on an Intel i7 platform, with 32 GB RAM, nVidia GeForce RTX5000, running on Ubuntu Linux 22.04 LTS.

In our experiments, we used the MobileNetV2 method. As the results show, it is suitable for edge computing deployments, and in our case, it achieved decent accuracy results.

The choice of the MobileNetV2 method is justified and explained by the research paper [20], where various models are compared to MobileNetV2 in terms of the mosaic images dataset used for training and inference. Another reason for selecting MobileNetV2 was that it was the fastest way to demonstrate that the video mosaicing method works well and is suitable for bird behavior recognition. Additionally, since behavior analysis requires processing large amounts of data with deep neural networks, we created 1,368,500 mosaic images from a single 5-min video for training. The dataset included mosaic images in five matrix variations and four frame-rate variations. The computations took two weeks on a powerful computer setup [20]. Using larger, resource-demanding deep neural network architectures was impractical for achieving the results. However, this could be included as a goal for future research.

The following configuration has been set up for chicken behavior recognition systems: a MobileNetV2 backbone model trained for a maximum of 20 epochs on a video sequence of chickens recorded by a CCTV camera—13 chickens annotated at a farm in The Netherlands. The output is in the form of mosaic images used for behavior classifications (see Table 1). The rationale behind selecting 13 chickens where the video showed 15 chickens was that only 13 remained in the FOV consistently, while two briefly disappeared and then returned to the CCTV viewpoint. This momentary disappearance signaled to CVAT that the object (chicken) was not present, and upon re-appearance, CVAT was unable to assign the same ID to this individual chicken. This edge case could be addressed on the side of our algorithm, if required.

### 3.5. Analysis of the Results

The chicken behavior recognition model performed very well, as we achieved more than 79.61% accuracy at the inference stage. The whole system training was designed to withstand different variations of splits of chicken IDs between training and testing sets. This led us to create custom folds, where all combinations of chicken IDs belonging to training or testing sets, were created (see Table 2).

The complete results of the classification with other details can be found in the Table 3.

**Figure 7:**
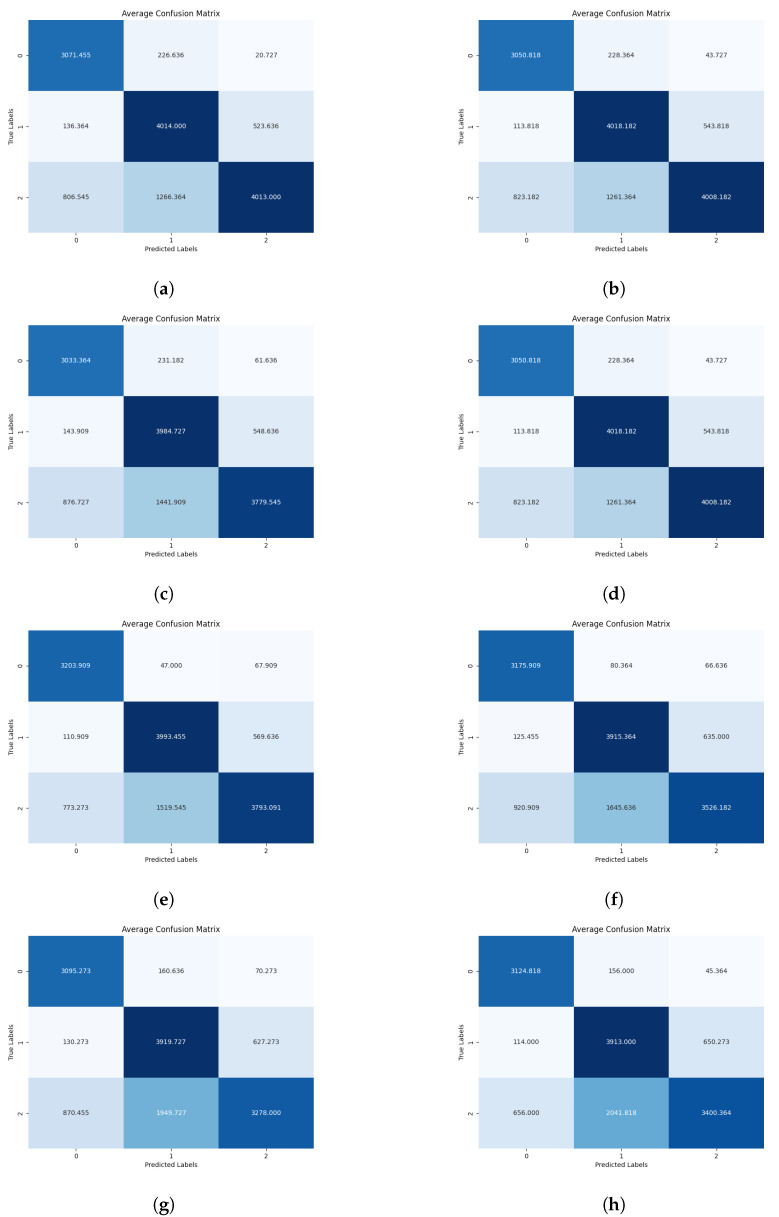
Diagrams showing combined confusion matrices across the 10 folds featuring specific mosaic configurations for 1, 2, 10, and 20 FPS sampling rates, where label 0 = Drinking, label 1 = Eating, label 2 = Sleepy. (**a**) Mosaic 2 × 2, 1 FPS. (**b**) Mosaic 2 × 2, 2 FPS. (**c**) Mosaic 2 × 2, 10 FPS. (**d**) Mosaic 2 × 2, 20 FPS. (**e**) Mosaic 3 × 3, 1 FPS. (**f**) Mosaic 3 × 3, 2 FPS. (**g**) Mosaic 3 × 3, 10 FPS. (**h**) Mosaic 3 × 3, 20 FPS.

**Figure 8:**
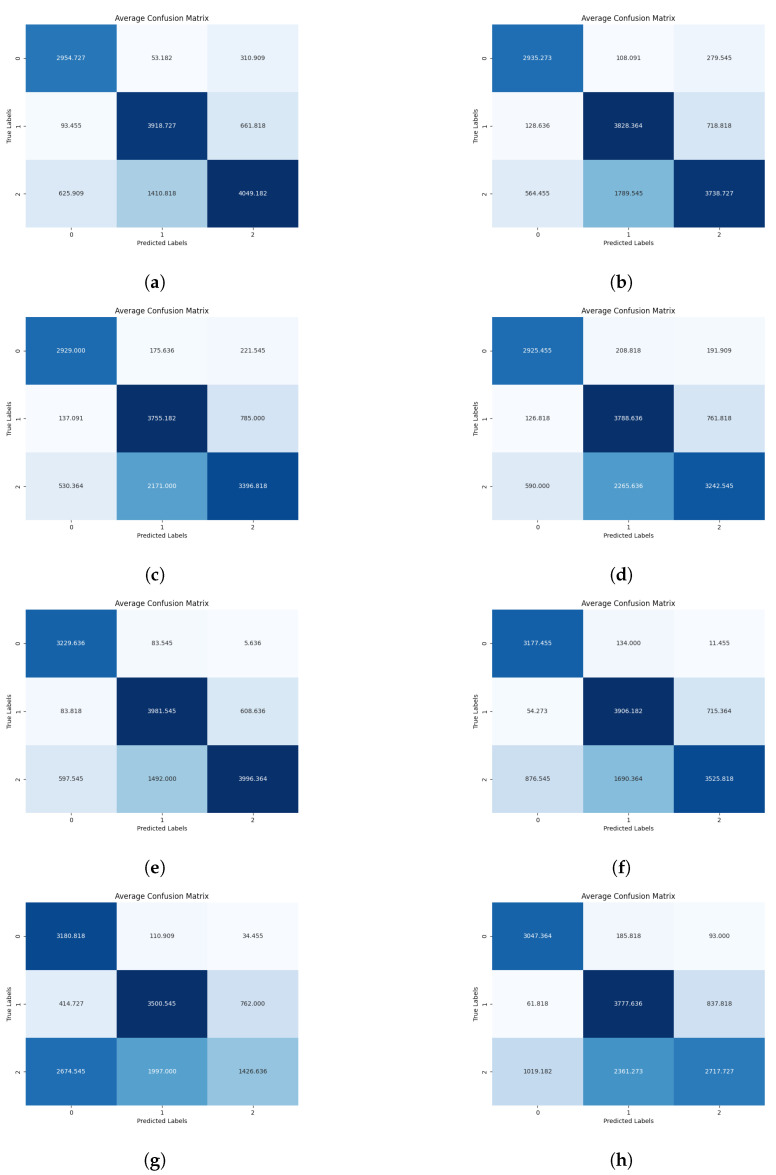
Diagrams showing combined confusion matrices across the 10 folds featuring specific mosaic configurations for 1, 2, 10, and 20 FPS sampling rates, where label 0 = Drinking, label 1 = Eating, label 2 = Sleepy. (**a**) Mosaic 4 × 4, 1 FPS. (**b**) Mosaic 4 × 4, 2 FPS. (**c**) Mosaic 4 × 4, 10 FPS. (**d**) Mosaic 4 × 4, 20 FPS. (**e**) Mosaic 5 × 5, 1 FPS. (**f**) Mosaic 5 × 5, 2 FPS. (**g**) Mosaic 5 × 5, 10 FPS. (**h**) Mosaic 5 × 5, 20 FPS.

**Figure 9:**
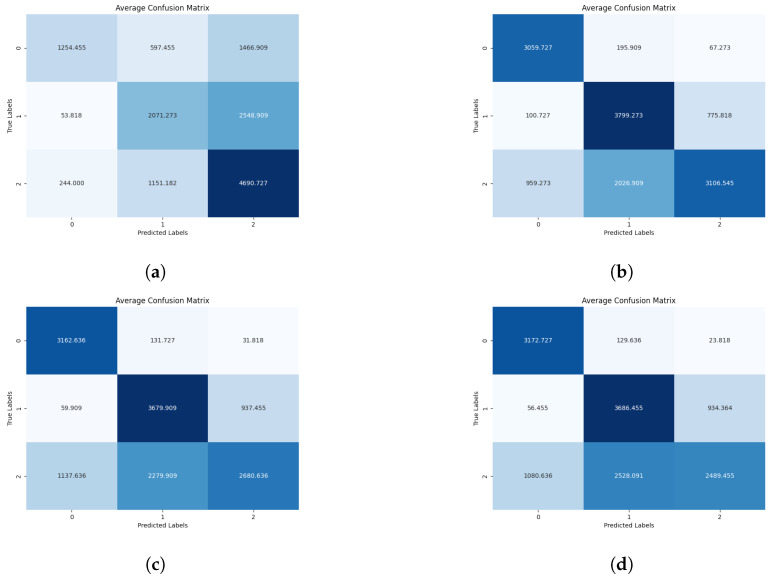
Diagrams showing combined confusion matrices across the 10 folds featuring specific mosaic configurations for 1, 2, 10, and 20 FPS sampling rates, where label 0 = Drinking, label 1 = Eating, label 2 = Sleepy. (**a**) Mosaic 6 × 6, 1 FPS. (**b**) Mosaic 6 × 6, 2 FPS. (**c**) Mosaic 6 × 6, 10 FPS. (**d**) Mosaic 6 × 6, 20 FPS.

At the inference stage, providing classification results for chicken behavior as the output, confusion matrices depicting probabilities of behaviors are generated (see Figure 7, Figure 8 and Figure 9).

Speaking of that, various videos have different bit-rates. In case of the CCTV video footage from the chicken cage taken in The Netherlands, the sampling rate was 20 fps, meaning that if we needed to have lower rates, we had to convert all frames to mosaic images based on this factor. In fact, with a frame rate of 1 frame per second, the mosaic images in the 5 × 5 format correspond to 25 s of video, and the 6 × 6 format corresponds to 36 s of video.

**Figure 10:**
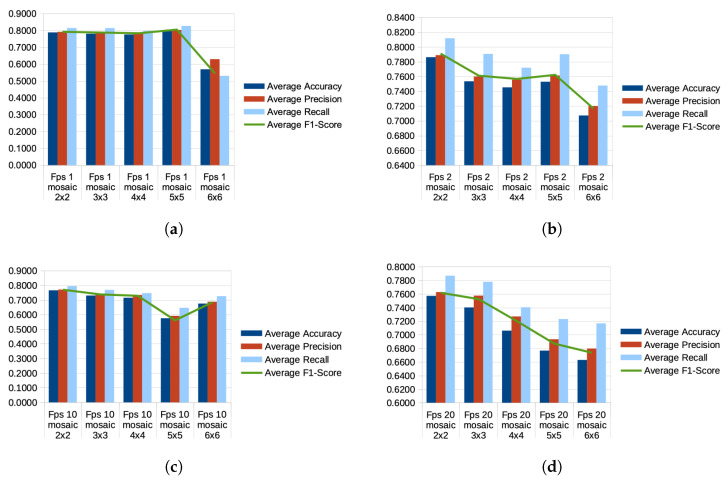
Diagrams show Average Accuracy, Average Precision, Average Recall, and Average F1-Score of Various Sampling Rates of the Various Mosaic Configurations. (**a**) Mosaic Split Configurations at 1 FPS. (**b**) Mosaic Split Configurations at 2 FPS. (**c**) Mosaic Split Configurations at 10 FPS. (**d**) Mosaic Split Configurations at 20 FPS.

Among the various mosaic combinations tested at a sampling rate of 1 fps, the best-performing split was 5 × 5, achieving an average accuracy of 79.61% across all folds. This was followed by 2 × 2 with 78.83% accuracy, then 3 × 3 (78.06%), 4 × 4 (77.58%), and finally, the lowest accuracy was observed with the 6 × 6 split, reaching 56.94% (see Figure 10a).

At a sampling rate of 2 fps, the best-performing mosaic variant was 2 × 2, with an average accuracy of 78.61% across all folds. This was followed by the 3 × 3 variant with 75.35% accuracy, then the 5 × 5 split with 75.29% accuracy, and finally, the 4 × 4 split with 74.53% accuracy (see Figure 10b).

When the sampling rate was increased to 10 fps, the best-performing mosaic split was 2 × 2, achieving 76.57% accuracy. Following this, the 3 × 3 split in mosaic images achieved 72.99% accuracy, closely followed by the 4 × 4 split with 71.49% accuracy. The 6 × 6 split performed slightly lower with 67.53% accuracy, while the worst-performing split was 5 × 5, with 57.50% accuracy, suggesting that the neural network struggled to distinguish behavioral information of chickens at this configuration (see Figure 10c).

At a sampling rate of 20 fps, equivalent to the original CCTV video footage, the best-performing mosaic split was 2 × 2, with an accuracy of 75.72%. This was followed by a consistent decline in accuracy for the 3 × 3 split (74.02% accuracy), the 4 × 4 split (70.61% accuracy), the 5 × 5 split (67.67% accuracy), and, finally, the 6 × 6 split with the lowest accuracy of 66.29% for this sampling rate (see Figure 10d).

**Figure 11:**
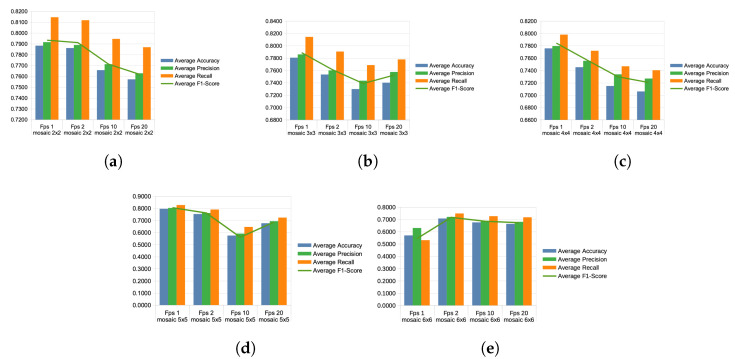
Diagrams showing Average Accuracy, Average Precision, Average Recall, and Average F1-Score of Various Sampling Rates of the Mosaic Configurations. (**a**) FPS 1, 2, 10, and 20, with Mosaic 2 × 2 configuration. (**b**) FPS 1, 2, 10, and 20, with Mosaic 3 × 3 configuration. (**c**) FPS 1, 2, 10, and 20, with Mosaic 4 × 4 configuration. (**d**) FPS 1, 2, 10, and 20, with Mosaic 5 × 5 configuration. (**e**) FPS 1, 2, 10, and 20, with Mosaic 6 × 6 configuration.

The best variable combination for the 2 × 2 division of mosaic images, with a sampling rate of 1 fps, emerged as the overall winner, achieving an average accuracy of 78.83% across the folds. However, as the fps sampling rates increased, the accuracy declined to 78.61% for 2 fps, further declining to 76.57% for the 10 fps combination, and ultimately to 75.72% for the 20 fps sampling. This mosaic configuration achieved an overall accuracy of 77.43% across all sampling rates (see Figure 11a).

The mosaic version of 3 × 3 showed less accuracy, with an average accuracy of 78.06% for 1 fps across 10 combined folds, which declined to 75.35% for 2 fps, further dropping to 75.99%. However, for the 20 fps version, the average accuracy increased to 74.02%. This suggests that this combination of faster sampling and mosaic division better interprets chicken movements than the previous 10 fps video sampling rate. This mosaic configuration achieved an accuracy of 75.11% across all sampling rates (see Figure 11b).

The graph illustrates the split of 4 × 4 mosaic images, which exhibited lower accuracy compared to the 3 × 3 variant. However, it showed the best performance in terms of accuracy at a 1 fps sampling rate, with an accuracy of 77.58%. Then, for 2 fps, the average accuracy declined to 74.53%, followed by a drop to 71.49% for 3 fps, and finally, the lowest average accuracy was observed for 20 fps, dropping to 70.61%. This mosaic configuration achieved an overall accuracy of 73.55% across all sampling rates (see Figure 11c).

This graph depicts different accuracies for the mosaic type 5 × 5, with 1 fps reaching 79.61%, then declining to 75.29% for 2 fps. However, for the 10 fps version, it reached the lowest level among all at 57.50%, but for the 20 fps configuration, it increased to 67.67%. This increase may indicate that the type of movements and behavior of chickens are better captured with the 1 fps mosaic images configuration. Less accuracy was observed with 2 fps sampling, and the lowest average accuracy was achieved using a sampling rate of 10 fps. This mosaic configuration achieved an accuracy of 70.02% across all sampling rates (see Figure 11d).

For the 6 × 6 mosaic configuration, the worst-performing sampling rate was 1 fps, with an accuracy of 56.94%, while the best-performing average accuracy was observed for the 2 fps configuration, reaching 70.72%. Following this, the accuracy was 67.53% for 10 fps and 66.29% for 20 fps. The poor results for this mosaic configuration could be explained by a lack of details captured by the neural network. With six images placed in the final mosaic, each image’s information was reduced, leading to lower accuracy rates compared to other mosaic splits. This mosaic configuration achieved an accuracy of 65.37% across all sampling rates (see Figure 11e).

The comparison with already existing state-of-the-art results can be found in Table 4.

The input of the MobileNetV2 is a dataset of images depicting various behaviors, organized in a specific directory structure. Each directory is named after a behavior class. For example, for three classes, for mosaic division 5 × 5 and sampling rate 10 FPS, we used the following directories:

- ./datasets/behavior/mosaic5x5fps10/Drinking/;
- ./datasets/behavior/mosaic5x5fps10/Eating/;
- ./datasets/behavior/mosaic5x5fps10/Sleepy/.

The output of the MobileNetV2 is a set of probabilities, which we presented as confusion matrices and also in a table, showing the probabilities of each class at the inference stage. Although there are data showing probabilities during the training and validation stages, we did not include it as this information does not accurately represent the true performance of our model. Within the mosaic image, the behavior of a single chicken was determined based on its movements. These movements were reconstructed based on the chicken’s position over time and depicted in each individual mosaic sub-image, with one chicken image per position at a time. Then, each mosaic image, along with its corresponding behavior class, was used for training. One mosaic image always belongs to one chicken and always shows a single behavior of that chicken.

### 3.6. Challenges

However, owing to the diverse challenges posed by variations in lighting conditions, discrepancies in the quality of CCTV cameras, fluctuations in the number of chickens present in the videos, and the differing behavioral patterns exhibited by the chickens across these locations, a strategic decision was made. We opted to exclusively focus on studying and processing videos solely from a farm in The Netherlands (see Figure 6), recognizing the importance of maintaining a consistent and controlled dataset for our research objectives. This focused approach allows for a more precise examination of behavioral patterns within a specific context, contributing to the robustness and reliability of our findings.

The study of chicken behavior presented us with several challenges. One of the main difficulties was identifying chickens that had similar physical appearances and keeping track of them using their IDs when they moved outside the FOV of the detection system.

The next challenge we faced was that the same chicken had a different appearance as it was growing. It is a relatively easy task for a human to recognize growing chicken, but it appeared to be a very difficult task for a deep neural network.

Another challenge arose when unregistered chickens entered the FOV, adding a layer of complexity to the analysis. Additionally, rearranging overlapping chickens within a flock proved to be a further obstacle.

Furthermore, occluded [45] chickens were observed near machinery, feeding equipment, or drinking pipes. Annotating their behavior became particularly intricate when chickens hid their heads either in their feathers or under the wing of another chicken.

Determining the appropriate annotation for a chicken transitioning from eating or drinking to taking a nap required thoughtful consideration. Deciding whether it still belonged to the ’Eating/Drinking’ category or shifted to the “Sleepy” category presented a unique challenge. These challenges underscore the intricacies we encountered in recognizing and annotating chicken behavior.

Last but not least, adopting the video mosaicing method for a different farm may introduce additional work as technical and environmental conditions most likely will be different from the farm that the system was trained for. This is in addition to different chickens that another chicken farm breeds.

**Figure 12:**
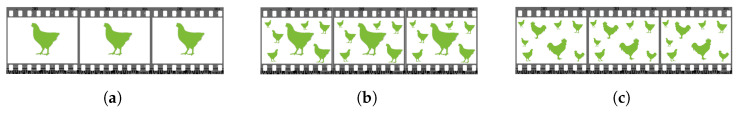
The diagrams show challenges which we had to overcome in order to process multiple instances of chickens in the same video sequence. The picture (**a**) shows single instance of one hen. (**b**) shows multiple instances of hens. (**c**) shows multiple instances of hens and roosters.

In this paper, while reviewing the method and developing software to support the research, we progressed from one instance of a hen in the video (Figure 12a) through multiple instances of hens (Figure 12b) to the final and most complex type of recognition with multiple instances of chickens (Figure 12c). The purpose of this work is to recognize activities, but not individual chickens. We also refrained from implementing the third recognition type due to the unavailability of video feeds. Nevertheless, our system is fully equipped and prepared to process this type of information when it becomes accessible. Tracking the behavior of multiple chickens in a video, especially when dealing with multiple instances of them, and this is also the case for other animals in general [46] and humans [47,48,49], is no easy task. To address this challenge, our software includes an array designed to keep track of the number of images used for each mosaic image creation. When the count reaches the array’s full capacity, as is the case with a 3 × 3 mosaic image configuration (totaling 9 images), a new mosaic image is generated for the specific chicken and saved in a directory corresponding to the chicken’s behavior. This process is contingent upon the unique chicken ID number assigned to the individual once it enters the camera’s field of view (FOV). The logic behind this assignment is rooted in the preceding annotation process.

The annotation of chicken behavior on the farm in The Netherlands, which is the case in our research, required keeping multiple chickens within the camera’s field of view (FOV). The camera was suspended from the top of the cage containing chickens, pointing towards the floor. The top-view of the chickens provided the least obstructed view of all individual chickens, except for two chickens that briefly went outside of the FOV. These two chickens were promptly disqualified from our research and removed from the annotations list.

## 4. Conclusions

This research contributes to the growing field of animal behavior recognition, specifically focusing on chickens, with implications for improved animal welfare practices, sustainable farming, and robotic vision applications. The number of recognized behavior types in chickens is anticipated to grow as veterinary specialists identify additional behaviors based on evolving needs and behavior analysis requirements. This expansion may encompass not only individual behaviors but also interactions between chickens, as well as between chickens and humans or other animals. This paper presents a comprehensive overview of the challenges faced, methodologies employed, and insights gained during the study. The results highlight the effectiveness and potential of our method for recognizing chicken behavior on edge computing devices, rendering it suitable for a wide range of applications. Continuously exploring and identifying different behavioral patterns will enrich our understanding of chicken behavior, thereby improving the breadth and precision of behavior analysis across various contexts. We conclude that recognizing chicken behavior through video mosaicing is an effective and valuable method for detecting sick chickens. It also serves to draw the attention of biologists and experts in the field to validate suspicions regarding the health of certain chickens through behavior analysis. This approach eliminates the need to treat all chickens with antibiotics regardless of their health status but allows for a focused treatment only on a few within the flock. Deploying methods on edge computing devices lacks practicality in our context, as our focus does not involve real-time systems and we do not incorporate novel behaviors in our approach. Additionally, we conclude that there are opportunities for future research, which could prove beneficial for both businesses and consumers.

Future development of the chicken behavior recognition system involves several key considerations for additional research and development. Firstly, there is a need to create distinct datasets that capture chicken behavior across different growth stages. This will enable the system to adapt and recognize varying behavioral patterns as chickens progress through different life phases. Additionally, efforts should be directed towards processing videos from different farms to generate farm-specific datasets. This approach will account for variations in camera types and lenses, ensuring the system’s adaptability to diverse farm environments. Expanding the range of behavior classes for chickens is essential, and involving specialists in behavior identification can contribute to more accurate and nuanced classifications. Moreover, exploring on-site processing is crucial to minimize the necessity for extensive image and video data transfers over the internet, promoting efficiency and data security. Testing video recording with automatic tilting and zooming capabilities represents an avenue for technological improvement. Striving for complete coverage (100%) of video recordings on chicken farms is an ambitious goal that can significantly enhance the system’s effectiveness. Simultaneously, efforts to enhance video data quality will contribute to improved chicken tracking accuracy, providing more reliable insights into their behavior.

For further improvements in the classification performance, we suggest increasing the quality and size of the behavior dataset. Additionally, implementing the system using transformer-type architectures based on the multi-head attention mechanism can enhance performance. Furthermore, incorporating multi-modality is expected to improve the performance of the behavior recognition system.

Looking beyond chickens, the future development of the system can extend its monitoring capabilities to various animals, including pigs, cows, and more. This expansion broadens the system’s applicability and further contributes to the field of animal behavior recognition in diverse agricultural settings. Insufficient real data, variability in behavior, and imbalance in classes, among other reasons, may require the use of synthetic data augmentation, which could also be considered in the future.

## Figures and Tables

**Table 1:** Experiments configuration set up for chicken behavior recognition. Legend: Ch. #(Chickens number), Test Acc. (Test Accuracy).

Model	Epochs	Input Data Farm	Input Format	Length	Output Format	Mosaic Images	Behavior Classes	Chickens	Test Acc.
MobileNetV2	20	The Netherlands	Video (MP4)	5 min	Mosaic, RGB	∼1,368,500	Sleepy, Eating, Drinking (3)	13	79.61%

**Table 2:** Chicken IDs split into folds used for training and testing.

Custom Folds	Chicken IDs Used for Training	Chicken IDs Used for Testing
C_Fold 0	3, 4, 5, 6, 7, 8, 9, 10, 11, 12	0, 1, 2
C_Fold 1	4, 5, 6, 7, 8, 9, 10, 11, 12, 0	1, 2, 3
C_Fold 2	5, 6, 7, 8, 9, 10, 11, 12, 0, 1	2, 3, 4
C_Fold 3	6, 7, 8, 9, 10, 11, 12, 0, 1, 2	3, 4, 5
C_Fold 4	7, 8, 9, 10, 11, 12, 0, 1, 2, 3	4, 5, 6
C_Fold 5	8, 9, 10, 11, 12, 0, 1, 2, 3, 4	5, 6, 7
C_Fold 6	9, 10, 11, 12, 0, 1, 2, 3, 4, 5	6, 7, 8
C_Fold 7	11, 12, 0, 1, 2, 3, 4, 5, 6, 7	8, 9, 10
C_Fold 8	12, 0, 1, 2, 3, 4, 5, 6, 7, 8	9, 10, 11
C_Fold 9	0, 1, 2, 3, 4, 5, 6, 7, 8, 9	10, 11, 12

**Table 3:** Summary per variable (variable 1—mosaic image matrix split; variable 2—frames-per second (FPS) sampling of the video) of evaluated metrics from the MobileNetV2 method using the chicken behavior data from the farm in The Netherlands, showing Average Accuracy (Avg. Accuracy), Average Precision (Avg. Precision), Average Recall (Avg. Recall), and Average F1-Score (Avg. F1-Score).

Variable 1	Variable 2	Avg. Accuracy	Avg. Precision	Avg. Recall	Avg. F1-Score
mosaic 2 × 2	FPS 1	0.7883	0.7915	0.8146	0.7934
mosaic 2 × 2	FPS 2	0.7861	0.7889	0.8118	0.7913
mosaic 2 × 2	FPS 10	0.7657	0.7712	0.7946	0.7713
mosaic 2 × 2	FPS 20	0.7572	0.7628	0.7869	0.7620
mosaic 3 × 3	FPS 1	0.7806	0.7860	0.8143	0.7890
mosaic 3 × 3	FPS 2	0.7535	0.7601	0.7906	0.7614
mosaic 3 × 3	FPS 10	0.7299	0.7434	0.7687	0.7390
mosaic 3 × 3	FPS 20	0.7402	0.7576	0.7779	0.7527
mosaic 4 × 4	FPS 1	0.7758	0.7795	0.7980	0.7845
mosaic 4 × 4	FPS 2	0.7453	0.7556	0.7719	0.7570
mosaic 4 × 4	FPS 10	0.7149	0.7337	0.7468	0.7300
mosaic 4 × 4	FPS 20	0.7061	0.7269	0.7404	0.7207
mosaic 5 × 5	FPS 1	0.7961	0.8030	0.8272	0.8063
mosaic 5 × 5	FPS 2	0.7529	0.7614	0.7901	0.7625
mosaic 5 × 5	FPS 10	0.5750	0.5911	0.6462	0.5622
mosaic 5 × 5	FPS 20	0.6767	0.6934	0.7232	0.6873
mosaic 6 × 6	FPS 1	0.5694	0.6297	0.5306	0.5457
mosaic 6 × 6	FPS 2	0.7072	0.7200	0.7477	0.7171
mosaic 6 × 6	FPS 10	0.6753	0.6880	0.7257	0.6854
mosaic 6 × 6	FPS 20	0.6629	0.6798	0.7168	0.6738

**Table 4:** Taxonomy used in Bird Behavior Recognition state-of-the-art methods. Legend: YP (year published), PA (paper), MOD (model), FRA (frames number), FI (Fighting (%)), PE (Perching (%)), FE (Feeding (%)), SW (Swimming (%)), FL (Flying (%)), WA (Walking (%)), ST (Standing (%)), EA (Eating (%)), SWF (Swimming and Flapping Wings (%)), STF (Standing and Feeding (%)), FLF (Flying and Feeding (%)), SFW (Standing and Flapping Wings (%)), CR (Crouching (%)).

PA	MOD	FRA	FI	PE	FE	SW	FL	WA	SW	EA	SWF	STF	FLF	SFW	CR
CVPR [27,28]	I3D	8	60.00	62.96	95.83	87.50	76.62	32.50	46.67	68.29	-	-	-	-	-
CVPR [27,28]	I3D	16	56.00	64.81	100	85.94	81.82	57.50	46.67	68.29	-	-	-	-	-
CVPR [27,28]	I3D	32	52.00	68.52	100	82.81	87.01	52.50	51.11	60.98	-	-	-	-	-
CVPR [29,30]	ResNeXt-50	-	-	-	-	97.09	94.52	-	90.42	-	51.11	70.06	23.61	47.83	66.40
CVPR [29,31]	SE-ResNet-50	-	-	-	-	96.22	91.44	-	86.58	-	64.44	64.97	18.06	47.83	63.20
CVPR [29,31]	SE-ResNeXt-50	-	-	-	-	94.77	92.47	-	88.18	-	62.22	69.75	19.44	39.86	58.40
CVPR [29,30]	ResNeXt-101	-	-	-	-	95.64	92.29	-	90.10	-	73.33	76.43	41.67	41.67	74.40
CVPR [29,31]	SE-ResNet-101	-	-	-	-	95.93	92.64	-	87.86	-	71.11	74.84	27.78	27.78	71.20
CVPR [29,31]	SE-ResNeXt-101	-	-	-	-	97.09	94.18	-	86.74	-	73.33	67.83	8.33	8.33	66.40
CVPR [27,32]	Non-Local	8	40.00	77.78	100	93.75	87.01	57.50	57.78	63.41	-	-	-	-	-
CVPR [27,32]	Non-Local	16	52.00	75.93	100	93.75	90.91	47.50	55.56	63.41	-	-	-	-	-
CVPR [27,32]	Non-Local	32	48.00	85.19	100	98.44	89.61	57.50	48.89	63.41	-	-	-	-	-
ECCV [29,33]	CBAM50	-	-	-	-	95.64	93.32	-	87.54	-	71.11	67.83	30.56	47.10	68.80
ECCV [29,33]	CBAM101	-	-	-	-	96.22	90.24	-	89.78	-	80.00	74.52	30.56	30.56	67.20
ICCV [27,34]	SlowFast	8 + 8	52.00	85.19	100	89.06	80.52	47.50	60.00	60.98	-	-	-	-	-
ICCV [27,34]	SlowFast	8 + 16	52.00	81.48	95.83	90.63	92.21	52.50	53.33	58.54	-	-	-	-	-
ICCV [27,34]	SlowFast	8 + 32	52.00	75.93	100	89.06	90.91	60.00	57.78	60.98	-	-	-	-	-
ICCV [27,35]	TSM	8	64.00	83.33	91.67	95.31	80.52	45.00	68.89	48.78	-	-	-	-	-
ICCV [27,35]	TSM	16	68.00	83.34	87.50	96.88	83.12	47.50	66.67	48.78	-	-	-	-	-
ICCV [27,35]	TSM	32	56.00	85.19	95.83	95.88	90.91	57.50	64.44	51.22	-	-	-	-	-
ACCV [29,36]	SGE-ResNet-50	-	-	-	-	95.06	93.66	-	86.90	-	60.00	72.29	18.06	52.90	67.20
ACCV [29,36]	SK-ResNet-50	-	-	-	-	96.22	93.15	-	88.34	-	73.33	75.16	25.00	50.00	61.60
IEEE-TPAMI [29,37]	Res2Net-50	-	-	-	-	96.22	93.15	-	89.14	-	64.44	68.15	23.61	49.28	64.80
IEEE-PAMI [29,38]	HRNet-w32	-	-	-	-	96.51	91.78	-	86.74	-	66.67	76.75	27.78	68.84	69.60
ACCV [29,36]	SGE-ResNet-101	-	-	-	-	95.93	94.01	-	86.58	-	68.89	76.11	25.00	25.00	69.60
ACCV [29,36]	SK-ResNet-101	-	-	-	-	96.22	94.01	-	91.05	-	77.78	73.25	25.00	25.00	64.80
IEEE [29,37]	Res2Net-101	-	-	-	-	95.06	92.29	-	91.21	-	75.56	66.88	31.94	31.94	68.00
IEEE-PAMI [29,38]	HRNet-w48	-	-	-	-	95.35	93.32	-	90.26	-	88.89	67.83	26.39	26.39	77.60
ICLR [27] [39]	V4D	8	40.00	88.89	100	89.06	80.52	52.50	60.00	63.41	-	-	-	-	-
ICLR [27,39]	V4D	16	44.00	92.59	100	92.19	79.22	50.00	60.00	63.41	-	-	-	-	-
ICLR [27,39]	V4D	32	48.00	83.33	100	90.63	80.52	67.50	62.22	63.41	-	-	-	-	-
CVPR [27,40]	TPN	8	44.00	85.19	100	100	90.91	52.50	71.11	58.54	-	-	-	-	-
CVPR [27,40]	TPN	16	40.00	92.59	100	100	90.91	57.50	53.33	63.41	-	-	-	-	-
CVPR [27,40]	TPN	32	52.00	81.48	100	100	88.31	70.00	48.89	60.98	-	-	-	-	-
CVPR [29,41]	SCNet50	-	-	-	-	95.06	92.12	-	85.94	-	68.89	68.47	22.22	50.72	67.20
IEEE-CVF [29,42]	ResNeSt-50	-	-	-	-	97.38	91.44	-	90.58	-	80.00	78.03	30.56	56.52	65.60
IEEE-CVF [29,41]	SCNet101	-	-	-	-	95.58	95.21	-	84.66	-	82.22	71.34	19.44	19.44	63.20
IEEE-CVF [29,42]	ResNeSt-101	-	-	-	-	97.38	92.64	-	92.81	-	75.56	79.30	37.50	37.50	72.00
ICCV [27,43]	MViT	8	56.00	90.74	100	95.31	80.52	47.50	51.11	65.85	-	-	-	-	-
ICCV [27,43]	MViT	16	52.00	81.48	100	100	76.62	40.00	48.89	63.41	-	-	-	-	-
ICCV [27,43]	MViT	32	60.00	83.33	100	90.63	89.61	37.50	57.78	60.98	-	-	-	-	-
NeurIPS [27,44]	TCP	8	76.00	75.93	100	79.69	87.01	57.50	73.33	58.54	-	-	-	-	-
NeurIPS [27,44]	TCP	16	72.00	74.07	91.67	90.63	92.21	65.00	60.00	56.10	-	-	-	-	-
NeurIPS [27,44]	TCP	32	60.00	79.63	70.83	82.81	90.91	50.00	73.33	68.29	-	-	-	-	-
EI-135 [29]	KFENet+-LFENet-w32	-	-	-	-	96.22	92.29	-	93.13	-	84.44	82.80	37.50	68.84	79.20
EI-135 [29]	KFENet+-LFENet-w48	-	-	-	-	96.22	92.00	-	93.45	-	86.67	82.17	48.61	67.39	80.00
EI-141 [27]	DF2-Net	8	56.00	79.63	100	93.75	90.91	62.50	71.11	56.10	-	-	-	-	-
EI-141 [27]	DF2-Net	16	44.00	94.44	100	98.44	93.51	75.00	53.33	63.41	-	-	-	-	-
EI-141 [27]	DF2-Net	32	56.00	83.33	100	98.44	92.21	65.00	42.22	58.54	-	-	-	-	-

## Data Availability

The code and datasets are available on request to the corresponding authors.
